# Ultrastructural analysis of apoptosis and autophagy in the midgut epithelium of *Piscicola geometra* (Annelida, Hirudinida) after blood feeding

**DOI:** 10.1007/s00709-015-0774-9

**Published:** 2015-02-10

**Authors:** M. M. Rost-Roszkowska, P. Świątek, I. Poprawa, W. Rupik, E. Swadźba, M. Kszuk-Jendrysik

**Affiliations:** Department of Animal Histology and Embryology, University of Silesia, Bankowa 9, 40-007 Katowice, Poland

**Keywords:** Midgut epithelium, Digestive system, Ultrastructure, Leech, Cell death

## Abstract

Cell death in the endodermal region of the digestive tract of the blood-feeding leech *Piscicola geometra* was analyzed using light and transmission electron microscopes and the fluorescence method. Sexually mature specimens of *P. geometra* were bred under laboratory conditions and fed on *Danio rerio*. After copulation, the specimens laid cocoons. The material for our studies were non-feeding juveniles collected just after hatching, non-feeding adult specimens, and leeches that had been fed with fish blood (*D. rerio*) only once ad libitum. The fed leeches were prepared for our studies during feeding and after 1, 3, 7, and 14 days (not sexually mature specimens) and some weeks after feeding (the sexually mature). Autophagy in all regions of the endodermal part of the digestive system, including the esophagus, the crop, the posterior crop caecum (PCC), and the intestine was observed in the adult non-feeding and feeding specimens. In fed specimens, autophagy occurred at very high levels—in 80 to 90 % of epithelial cells in all four regions. In contrast, in adult specimens that did not feed, this process occurred at much lower levels—about 10 % (esophagus and intestine) and about 30 % (crop and PCC) of the midgut epithelial cells. Apoptosis occurred in the feeding adult specimens but only in the crop and PCC. However, it was absent in the non-feeding adult specimens and the specimens that were collected during feeding. Moreover, neither autophagy nor apoptosis were observed in the juvenile, non-feeding specimens. The appearance of autophagy and apoptosis was connected with feeding on toxic blood. We concluded that autophagy played the role of a survival factor and was involved in the protection of the epithelium against the products of blood digestion. Quantitative analysis was prepared to determine the number of autophagic and apoptotic cells.

## Introduction

During digestion in blood-feeding animals, the blood generates many molecules which may be toxic or even lethal for the organism (Dunkov et al. 2002; Taketani 2005). Therefore, several mechanisms which neutralize the toxic molecules must be activated in order to maintain the homeostasis of the organism (Okuda et al. 2005, 2007). Toxic substances can accumulate in numerous structures called spherites (Oliveira et al. 2000; Lipovšek et al. 2002, 2012) or in vacuoles called endosomes (Tarnowski and Coons 1989). The perimicrovillar or the peritrophic membranes, which separate the midgut epithelium from the midgut lumen, protect the organism against the entrance of pathogens or toxic substances (Terra 2001; Albuquerque-Cunha et al. 2004; Terra and Ferreira 2012). Another protective mechanism is the accumulation of endosymbionts in the midgut epithelium or even in specialized organs called mycetomes (Perkins et al. 2005; Graf et al. 2006). However, disrupted cells or cells with many toxic molecules can lead to cell death. Eventually, they can be discharged from the midgut epithelium into its lumen. As a consequence, such cells will not affect the entire epithelium and the organism will be protected against the effects of stress factors (e.g., toxic substances) (Franzetti et al. 2012; Rost-Roszkowska et al. 2012b; Wilczek et al. 2014). These cells before being removed into the extracellular space or midgut lumen must die in one of the kinds of cell death. Many types of the cell death have been described in animals (e.g., apoptosis, necrosis, autophagy, anoikis, entosis, necroptosis, senescence, or a mitotic catastrophe) as processes which participate in homeostatic maintenance (Proskuryakov et al. 2003; Overholtzer et al. 2007; Vanden Berghe et al. 2010; Rost-Roszkowska et al. 2010a, b, 2012b, 2013; Franzetti et al. 2012; Klionsky et al. 2012). However, mainly apoptosis, necrosis, and autophagy have been distinguished in the midgut epithelium of invertebrates (Takeda et al. 2001; Uwo et al. 2002; Martins et al. 2005; Wu et al. 2006; Tettamanti et al. 2007a, b; Park and Takeda 2008; Rost-Roszkowska et al. 2008, 2010a, b, 2012b, 2013; Teixeira et al. 2013; Wilczek et al. 2014).

The fish leech *Piscicola geometra* is a widespread ectoparasite that attaches to the body, gills, or mouth of numerous fish species, e.g., salmon (*Salmo salar*), brown trout (*Salmo trutta*), charr (*Salvelinus alpinus*), bream (*Abramis brama*), perch (*Perca fluviatilis*), gibel carp (*Carassius gibelio*), and the common carp (*Cyprinus carpio*) (Arslan and Emiroğlu 2011). It feeds on the blood which may accumulate in the leeches digestive system for some months. The precise ultrastructure of the midgut of *P. geometra* with special emphasis on blood accumulation and the role of endosymbionts was described in our previous article (Rost-Roszkowska et al. 2012a). However, when analyzing the organization of the endodermal region of the digestive system of *P. geometra*, we observed signs of cell death. Therefore, the aims of this study were (1) to study which kind(s) of cell death occur(s) in non-feeding juveniles, non-feeding adults, and feeding adult specimens; (2) to analyze the relationship between cell death and the time after feeding; (3) to describe cell death at the ultrastructural level; and finally, (4) to state whether cell death participates in the neutralization of toxic substances which originate from blood.

## Material and methods

### Material

Sexually mature specimens of *P. geometra* (Linnaeus, 1758) were collected from *C. carpio* (commercial culture) and were bred under laboratory conditions in 10,000 cm^3^ aquaria. They were fed on *Danio rerio*. Two weeks after the copulation, the specimens laid cocoons and died. The material for our studies was non-feeding juveniles collected just after hatching (they did not have the possibility to feed), non-feeding adult specimens (they did not feed for 5–6 weeks), adult specimens during feeding (they were collected from *D. rerio* bodies), and leeches that had been fed with fish blood (*D. rerio*) only once ad libitum. After the blood meal, the fish were removed from the aquarium. The fed leeches were prepared for our studies after 1, 3, 7, and 14 days (not sexually mature specimens) and some weeks after feeding (these specimens achieved sexual maturity and were fixed just after copulation). In total, 4 juveniles and 54 adult specimens were collected, fixed, and studied using light, fluorescence, and transmission electron microscopy and also histochemical techniques. Principles of laboratory animal care were followed, as well as specific national laws where applicable. All specimens of *D. rerio* (Teleostei, Cyprinidae) (Hamilton-Buchanan 1822) were cultured at the Zoological Institute of Wrocław University according to approval of Commission for the Ethical Treatment of Animals in Wrocław (number 05.2008).

### Methods

#### Light and electron microscopy

The specimens studied were prepared in the manner decsribed for previously studied clitellate annelids (Rost-Roszkowska et al. 2012a; Świątek et al. 2012). The bodies of 29 adult specimens: two non-feeding adult specimens, two adult specimens during feeding (collected from *D. rerio* bodies), and for five specimens collected 1, 3, 7, and 14 days after feeding (not sexually mature specimens), five specimens collected some weeks after feeding (sexually mature specimens after copulation), and additionally three juvenile non-feeding specimens, were fixed in 2.5 % glutaraldehyde in a 0.1 M sodium phosphate buffer (pH 7.4) at room temperature (RT) for 2 days. After washing in a sodium phosphate buffer, the material was postfixed for 2 h in 1 % OsO_4_ in the same buffer, dehydrated in a graded series of ethanol (50, 70, 90, 95, and 100 %, each for 15 min), transferred to acetone (15 min), and embedded in epoxy resin (Epoxy Embedding Medium Kit; Sigma, St. Louis, MO). Semithin sections (0.8 μm thick) after staining with 1 % methylene blue in 0.5 % borax were examined under an Olympus BX60 microscope (LM). Ultra-thin sections (80 nm) were cut on a Leica Ultracut UCT Ultramicrotome. After staining with uranyl acetate and lead citrate, the sections were examined using a Hitachi H500 electron microscope at 75 kV (TEM).

Additionally, semithin sections of all regions of the midgut (esophagus, crop, posterior crop caecum, intestine) were used in order to count the number of epithelial cells with morphological signs of autophagy and apoptosis in relation to the total number of cells. The percentage of autophagic and apoptotic cells was determined by randomly counting 100 cells.

#### Histochemistry

The pieces of the bodies of the 25 adult specimens (for five specimens at 1, 3, 7, and 14 days after feeding and five specimens after copulation) and 1 juvenile non-feeding specimen that were analyzed were fixed in 4 % paraformaldehyde in Tris-buffered saline (TBS) for 20 min at room temperature, placed in TBS containing 0.1 % Triton X-100 and embedded in a tissue-freezing medium (Jung). Cryostat sections were cut (5 μm of thickness) and placed on 1 % gelatin-coated slides.

#### Acid phosphatase staining

After the cryosections were washed in TBS (5 min, RT) and a 0.1 M sodium acetate-acetic acid buffer (pH 5.0–5.2, RT), the material was incubated in a 0.1 M sodium acetate-acetic acid buffer pH 5.0–5.2 containing 0.01 % naphthol phosphate AS-BI, 2 % *N-N*-dimethylformamide, 0.06 % Fast Red Violet LB, and 0.5 mM MnCl_2_ (1.5 h, 37 °C). Slides were analyzed using an Olympus BX60 light microscope (LM).

#### TUNEL assay

Cryostat sections were incubated in a permeabilization solution (0.1 % sodium citrate) (2 min on ice in 4 °C), washed in TBS (3 × 5 min) and stained with a terminal deoxynucleotidyl transferase dUTP nick end labeling (TUNEL) reaction mixture (In Situ Cell Death Detection Kit, TMR red, Roche; 60 min at 37 °C in the dark). Negative controls were prepared according to the labeling protocol (In Situ Cell Death Detection Kit, TMR red, Roche). Slides were analyzed using an Olympus BX60 fluorescence microscope (FM).

## Results

The midgut (endodermal region of the digestive tract) in juvenile and adult specimens of *P. geometra* is composed of four regions: the esophagus, the crop, the posterior crop caecum (PCC), and the intestine. Its epithelium is formed by two kinds of cells: digestive and small cells of an unknown function. The precise ultrastruture of the midgut epithelium was described in a previous article (Rost-Roszkowska et al. 2012a).

### Autophagy

**Fig. 1:**
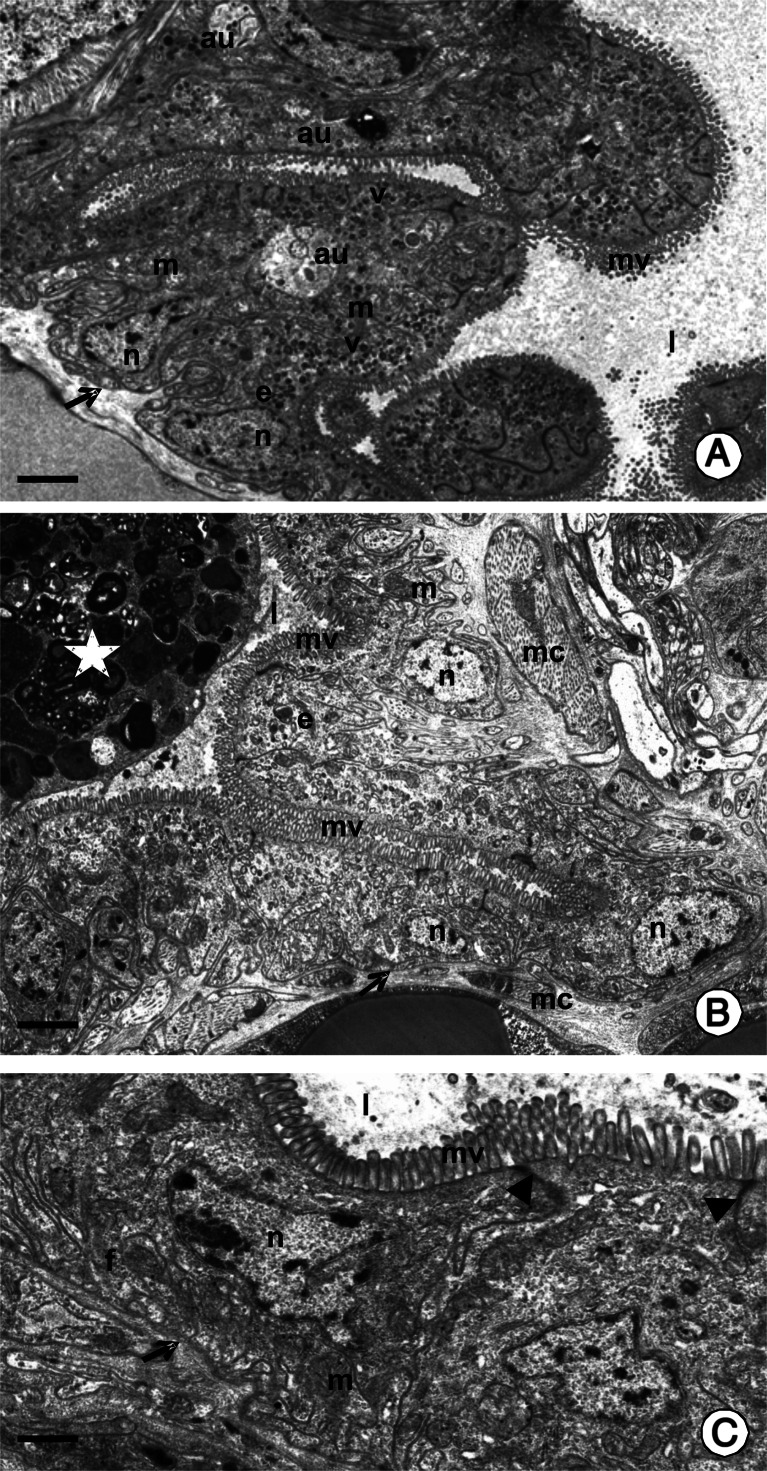
TEM images of autophagy in the midgut epithelium of *P. geometra. e* midgut epithelium, *l* midgut lumen, *mv* microvilli, *m* mitochondria, *n* nucleus, *v* electron-dense vesicles, *mc* visceral muscles, *f* folds of the basal cell membrane. **a** The epithelium of the crop of adult feeding specimen with signs of autophagy. Specimen analyzed 14 days after feeding. *Arrow* basal lamina. *Bar* = 1.4 μm. **b** The epithelium of the crop in juvenile, non-feeding specimen. *Star* remnants of the embryonic nutritive material; *arrow* basal lamina. *Bar* = 1.5 μm. **c** The epithelium of the posterior crop caecum (*PCC*) in juvenile, non-feeding specimen. *Arrowhead zonulae adherens* between adjacent epithelial cells; *arrow* basal lamina. *Bar* = 0.6 μm

**Fig. 2:**
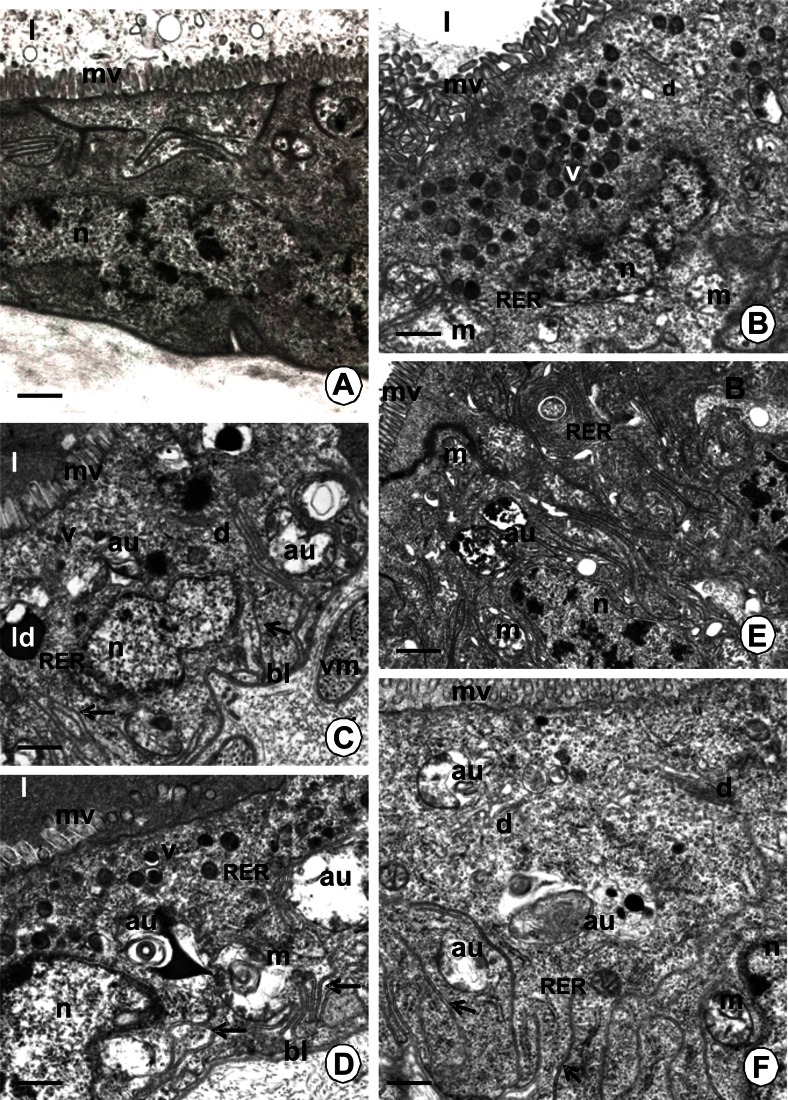
TEM images of autophagy in the midgut epithelium of *P. geometra. au* autophagosome, *mv* microvilli, *l* midgut lumen, *n* nucleus, *m* mitochondria, *v* electron-dense vesicles, *d* Golgi complexes, *RER* cisterns of the rough endoplasmic reticulum, *ld* lipid droplet, *bl* basal lamina. **a** The epithelium of esophagus in non-feeding adult specimens. *Bar* = 0.5 μm. **b** The epithelium of the crop in non-feeding adults. *Bar* = 0.8 μm. **c** The epithelium of the crop in adult specimens of *P. geometra* collected during feeding. *Arrows* folds of the basal membrane. *Bar* = 0.5 μm. **d** The epithelium of PCC in adult specimens collected during feeding on fish blood. *Arrows* folds of the basal membrane. *Bar* = 0.3 μm. **e** The epithelium of the intestine in non-feeding adult specimens with signs of autophagy. *Bar* = 0.8 μm. **f** The epithelium of the intestine of adult specimen collected during feeding with signs of autophagy. *Arrows* folds of the basal membrane. *Bar* = 0.3 μm

The process of autophagy was observed in the adult non-feeding and feeding specimens (Table 1; Figs. 1a and 2a–f), while it was absent in the juvenile, non-feeding specimens (Table 1; Fig. 1b, c). It occurred in all regions of the endodermal part of the digestive system in the feeding adult specimens: the esophagus, the crop, the PCC, and the intestine (Table 1) as a common process (80–90 % cells showed signs of autophagy), and it was not dependent on the time after feeding (Table 2). When the adult specimens did not feed, this process occurred only in about 10 % of cells in the esophagus and intestine (Fig. 2e), while the majority of digestive cells did not show any signs of autophagy (Fig. 2a). Autophagy reached 30 % in the crop and PCC of unfed adults (Fig. 2b), considerably lower than the 80–90 % seen in fed adults.

**Table 1:** The comparison of autophagy and apoptosis appearance in non-feeding juveniles, non-feeding adults and feeding adult specimens of *Piscicola geometra*

	Esophagus	Crop	Posterior crop caecum	Intestine
Non-feeding juveniles				
Autophagy	−	−	−	−
Apoptosis	−	−	−	−
Non-feeding adult specimens				
Autophagy	+	+	+	+
Apoptosis	−	−	−	−
Adult feeding specimens				
Autophagy	+	+	+	+
Apoptosis	−	+	+	−

**Table 2:** The percentage of autophagic and apoptotic midgut epithelial cells in non-feeding juveniles, non-feeding and feeding adult specimens of *Piscicola geometra*

	Esophagus	Crop	Posterior crop caecum	Intestine
Non-feeding juveniles				
Autophagy	0 %	0 %	0 %	0 %
Mean	0	0	0	0
SE	0.00	0.00	0.00	0.00
Apoptosis	0 %	0 %	0 %	0 %
Mean	0	0	0	0
SE	0.00	0.00	0.00	0.00
Non-feeding adult specimens				
Autophagy	10 %	30 %	30 %	10 %
Mean	10	30	29.6	10.2
SE	0.37	0.37	0.37	0.29
Apoptosis	0 %	0 %	0 %	0 %
Mean	0	0	0	0
SE	0.00	0.00	0.00	0.00
Adult specimens collected during feeding				
Autophagy	60 %	60 %	60 %	60 %
Mean	59.4	60	59.7	59.6
SE	0.70	0.56	0.87	0.65
Apoptosis	0 %	0 %	0 %	0 %
Mean	0	0	0	0
SE	0.00	0.00	0.00	0.00
Adult feeding specimens collected 1 day after feeding				
Autophagy	80 %	80 %	80 %	80 %
Mean	80.1	80.3	79.7	80.9
SE	0.66	0.87	0.54	0.96
Apoptosis	0 %	10 %	5 %	0 %
Mean	0	0	0	0
SE	0.00	0.00	0.00	0.00
Adult feeding specimens collected 3 days after feeding				
Autophagy	90 %	90 %	90 %	90 %
Mean	90.6	90.9	91	90.6
SE	0.67	0.55	0.60	0.37
Apoptosis	0 %	12 %	8 %	0 %
Mean	0	0	0	0
SE	0.00	0.00	0.00	0.00
Adult feeding specimens collected 7 days after feeding				
Autophagy	90 %	90 %	90 %	90 %
Mean	90.9	90.5	90.1	89.3
SE	0.89	0.64	0.31	0.80
Apoptosis	0 %	15 %	10 %	0 %
Mean	0.00	15.10	9.70	0.00
SE	0.00	0.57	0.30	0.00
Adult feeding specimens collected 14 days after feeding				
Autophagy	90 %	90 %	90 %	90 %
Mean	90	91.3	90.6	90.4
SE	0.52	0.30	0.50	0.50
Apoptosis	0 %	20 %	15 %	0 %
Mean	0	20.1	14.8	0
SE	0.00	0.38	0.53	0.00
Adult feeding specimens collected after copulation				
Autophagy	90 %	90 %	90 %	90 %
Mean	91.3	90.1	90.1	90.2
SE	0.37	0.38	0.57	0.36
Apoptosis	0 %	25 %	20 %	0 %
Mean	0	25.3	20.2	0
SE	0.00	0.37	0.36	0.00

*SE* standard error

**Fig. 3:**
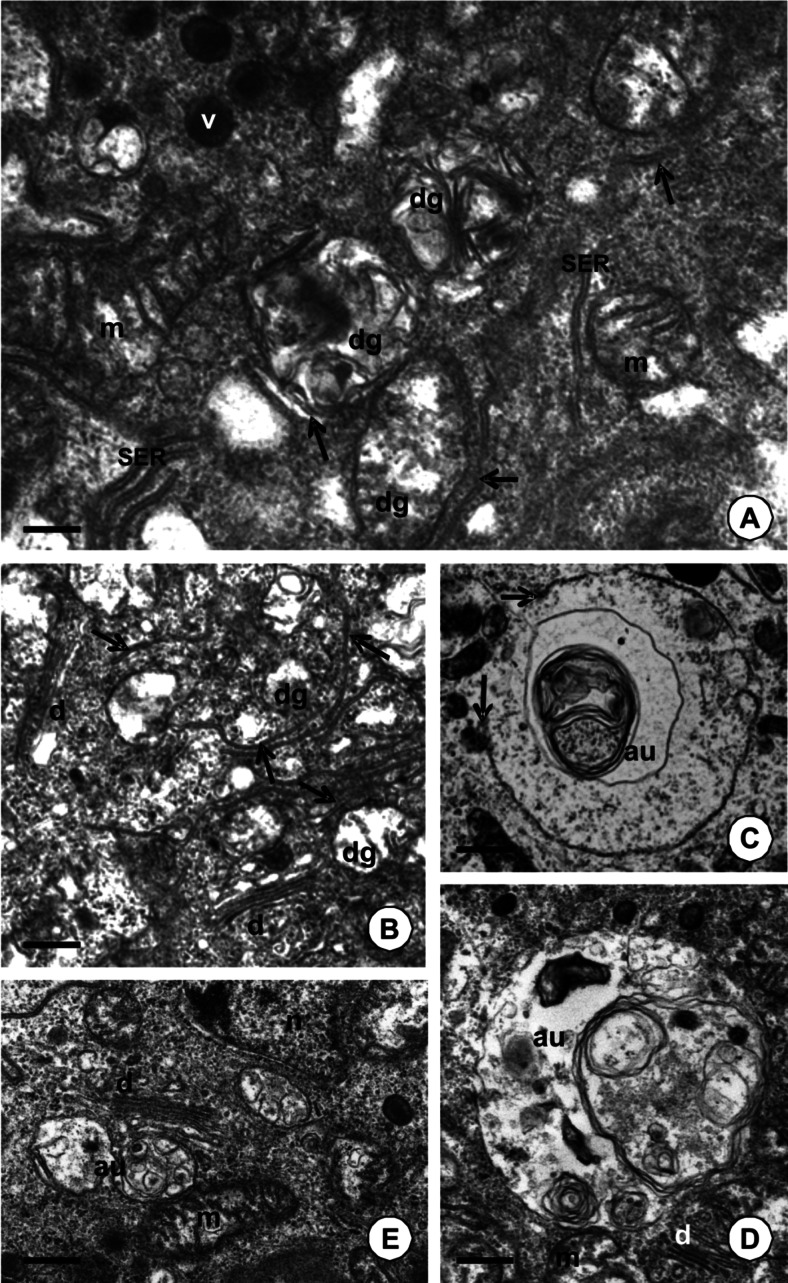
TEM images of autophagy in the midgut epithelium of adult feeding specimens of *P. geometra. m* mitochondria, *v* electron-dense vesicles, *au* autophagosome, *d* Golgi complexes, *n* nucleus. **a** Specimen analyzed 14 days after blood meal. Digestive cell of the crop. Cisterns of the smooth endoplasmic reticulum (*SER*) accumulate in the neighborhood of degenerated organelles (*dg*) and gradually surround them (*arrows*). *Bar* = 0.25 μm. **b** Specimen studied 1 day after feeding. Digestive cell of PCC. Golgi complexes accumulate in the neighborhood of degenerated organelles (*dg*) and gradually surround them (*arrows*). *Bar* = 0.35 μm. **c** Specimen analyzed after copulation. Digestive cell of the crop. Expanded membranes of phagophore (*arrows*). Autophagosome is gradually formed. *Bar* = 0.5 μm. **d** Specimen analyzed after copulation. Digestive cell of the crop. *Bar* = 0.3 μm. **e** Specimen studied 14 days after feeding. Digestive cell of the intestine. Golgi complexes accumulate in the neighborhood of autophagosomes. *Bar* = 0.4 μm

**Fig. 4:**
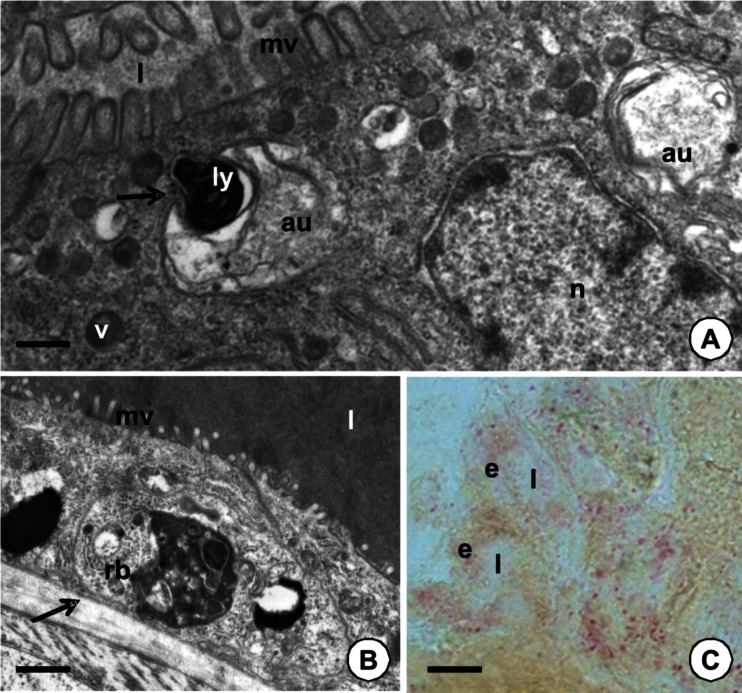
*l* midgut lumen, *mv* microvilli, *n* nucleus, *v* electron-dense vesicles, *e* midgut epithelium. **a** TEM image of midgut epithelium in adult specimen analyzed after copulation. Digestive cells of PCC. Fusion (*arrow*) of autophagosomes (*au*) with lysosome (*ly*). *Bar* = 0.25 μm. **b** TEM image of the midgut epithelium of specimen after copulation. Digestive cells of the crop. Residual bodies with electron-dense content (rb). Midgut lumen filled with digested blood (*l*); *arrow* basal lamina. *Bar* = 0.75 μm. **c** Positive reaction for acid phosphatase staining (*stained pink*) in specimen studied 7 days after feeding. *Bar* = 7.8 μm

The organelles which were targeted for degeneration accumulated in the neighborhood of the cisterns of the smooth endoplasmic reticulum and Golgi complexes (Fig. 3a, b). Autophagy began with the formation of the phagophore. Cisterns of flat cup-shaped membranes surrounded the engulfed regions of the cytoplasm together with degenerated organelles (e.g., mitochondria) (Fig. 3a, b). The edges of the phagophore expanded (Fig. 3c) and gradually the membraned autophagosomes were completely closed (Fig. 3d). However, numerous cisterns of Golgi complexes were observed in the neighborhood of the autophagosomes (Fig. 3d, e). Autolysosomes were formed after the fusion of the autophagosomes with lysosomes (Fig. 4a) and eventually residual bodies with an electron-dense content occurred (Fig. 4b). In several cases, when many large autophagosomes, autolysosomes, or residual bodies were accumulated in the cytoplasm, the apical cell membrane caused its invagination. After cell membrane breakage, the autophagosomes/autolysosomes/residual bodies were discharged into the midgut lumen (not shown). A positive reaction for acid phosphatase staining (Fig. 4c) confirmed the participation of this enzyme in autophagy.

### Apoptosis

**Fig. 5:**
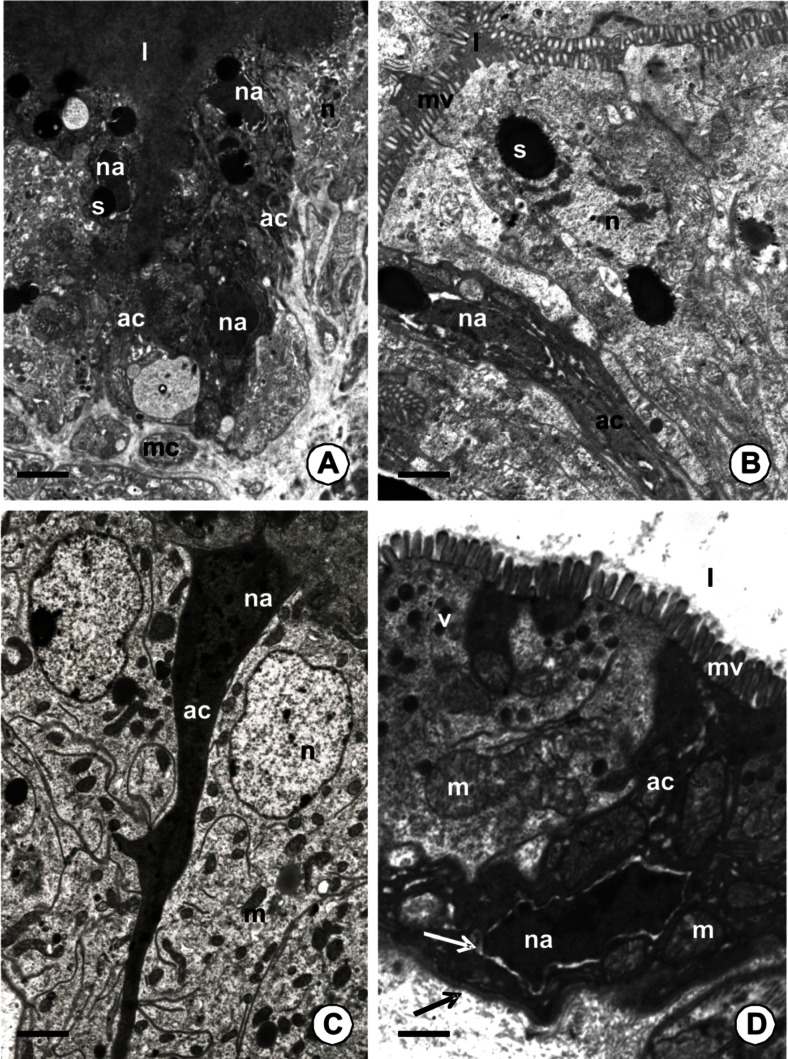
TEM images of apoptosis in the midgut epithelium of adult feeding specimens of *P. geometra. l* midgut lumen filled with digested blood, *na* nuclei of apoptotic cells, *n* nucleus of adjacent digestive cell, *mc* visceral muscles, *s* storage material, *ac* apoptotic cells, *m* mitochondria, *mv* microvilli, *v* electron-dense vesicles. **a** The epithelium of the PCC. In specimens 14 days after blood meal numerous apoptotic cells form groups. *Bar* = 2 μm. **b** Specimen studied 3 days after feeding. The epithelium of the PCC. Single apoptotic cell is observed. *Bar* = 1.1 μm. **c** Specimen after copulation. The epithelium of PCC. Electron-dense cytoplasm of apoptotic cell. *Bar* = 1 μm. **d** Specimen after copulation. The epithelium of the crop. Distinct space between the nucleus and the cytoplasm in apoptotic cell appears (*white arrow*). *Black arrow* basal lamina. *Bar* = 0.5 μm

Apoptosis was observed as a common process only in the adult feeding specimens of *P. geometra.* It was absent in non-feeding adult specimens, specimens that were collected during feeding, and in the juvenile, non-feeding specimens. Among all four parts of the endodermal part of the digestive system, it was observed only in the crop and PCC, but it was not found in the esophagus and intestine (Table 1). Apoptosis was mainly detected in the specimens that were studied 14 days after feeding (Table 2; Fig. 5a) and in the specimens after copulation, while it was a sporadic process in the specimens analyzed 1, 3, or 7 days after feeding (Table 2; Fig. 5b).

**Fig. 6:**
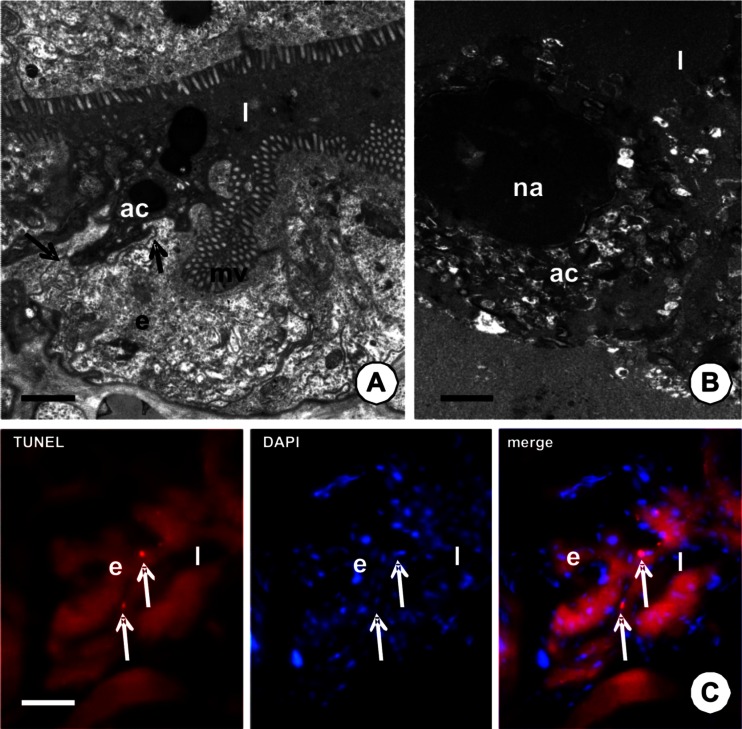
**a**–**b** TEM images of adult specimens of *P. geometra*—midgut epithelium*. e* midgut epithelium, *mv* microvilli, *ac* apoptotic cell, *l* midgut lumen, *na* nucleus of apoptotic cell. **a** Specimen 3 days after feeding. The crop. Apoptotic cell gradually discharged into the midgut lumen. Intercellular junctions (*arrows*) between digestive cells. *Bar* = 1 μm. **b** One day after feeding. The crop. The remnants of the apoptotic cell in the midgut lumen. *Bar* = 0.8 μm. **c** TUNEL-positive signals (*arrows*) in the midgut epithelium of the specimen 7 days after feeding. Nuclei of the midgut epithelium stained in blue (DAPI staining). *Bar* = 23.5 μm

The first morphological sign of the beginning of apoptosis was the shrinkage of the cytoplasm, which gradually became electron dense (Fig. 5a, c). Distinct extracellular spaces appeared between the apoptotic and adjacent digestive cells. The nucleus achieved a lobular and strongly irregular shape with the chromatin condensed near the nuclear envelope (Fig. 5d). Mitochondria lost their crista and the cisterns of endoplasmic reticulum started to swell. Distinct spaces appeared between the nucleus and the cytoplasm (Fig. 5d). In the process of the gradual shrinkage of the cells, the apoptotic cell was separated from the basal lamina. Intercellular junctions were formed between the basal and perinuclear regions of adjacent epithelial cells (Fig. 6a), while they were still present between the apical regions of apoptotic and adjacent cells. Eventually, the degenerating cells were discharged into the midgut lumen (Fig. 6a), where they underwent digestion (Fig. 6b). The formation of apoptotic bodies and phagocytosis was not observed. TUNEL-positive signals were observed in the nuclei of the digestive cells (Fig. 6c). The midgut epithelium that was incubated without a TdT enzyme solution showed no signals (not shown).

### Necrosis

Necrosis occurred sporadically only in adult specimens. Necrotic cells possessed electron-lucent cytoplasm. The apical membrane was disrupted, and the cytoplasm together with organelles were discharged into the midgut lumen (not shown).

## Discussion

During blood digestion in hematophagous invertebrates, many molecules which might be toxic for the entire organism are produced (Taketani 2005). This causes the activation of different mechanisms which take part in the neutralization of these toxic molecules (Graça-Souza et al. 2006; Okuda et al. 2007): the accumulation of hemoxisomes, spherocrystals, or electron-dense granules with iron, the formation of crystalline hemozoin, and the increased formation of the molecules of ferritin (Tarnowki and Coons 1989; Bradbury 1995; Oliveira et al. 2000; Dunkov et al. 2002; Silva et al. 2006; Filimonova 2008; Azevedo et al. 2009). Numerous vesicles with an electron-dense content that gather in the apical cytoplasm were observed in *P. geometra* (Rost-Roszkowska et al. 2012a). However, they were present in juvenile, non-feeding specimens and the content of such vesicles increased in electron density after feeding. Therefore, in our previous paper, we stated that they play a role in enzyme accumulation and not in the protection of the epithelium/midgut/organism against the toxic substances that originate from blood (Rost-Roszkowska et al. 2012a). Therefore, we wondered which mechanisms would protect the organism against the lethal effects of blood as a stress factor.

Cell death is responsible for homeostatic maintenance during both embryogenesis and throughout the entire life span of an animal. It controls the size of the cell population under both normal and pathological conditions, because it enables all of the cells which would activate the inflammatory condition to be eliminated from the tissue/organ (Kerr 2002; Baehrecke 2003; Kroemer et al. 2009; Tettamanti et al. 2011; Wilczek et al. 2014). Two main processes of cell death—apoptosis and autophagy—and the relationship between them are commonly studied at present. They are the mechanisms which enable disrupted and damaged cells to be removed from the organism. While apoptosis is a kind of programmed cell death, the turnover of damaged organelles by the phagophore and/or lysosomes might be treated as a survival factor (Lee and Baehrecke 2001; Chera et al. 2009; Kourtis and Tavernarakis 2009; Malagoli et al 2010; Klionsky et al. 2012; Park et al. 2013; Teixeira et al. 2013). In this case, autophagy can play the role of a survival factor by preventing the death of a cell or it can lead to self-digestion (Levine and Klionsky 2004; Giusti et al. 2007; Tettamanti et al. 2007a; Rost-Roszkowska et al. 2008, 2012b, 2013; Park et al. 2009; Malagoli et al. 2010; Park et al. 2013). Apoptosis and autophagy occurred in the adult specimens of *P. geometra* which were fed with fish blood, while they were not observed in the juvenile non-feeding specimens. However, autophagy was also detected in the non-feeding adults, but it did not occur as a common process (only 10 or 30 % of midgut epithelial cells showed signs of autophagy). In our previous paper, some data about the presence of sporadic autophagosomes/autolysosomes in the digestive cells in the crop of juvenile non-feeding specimens were provided (Rost-Roszkowska et al. 2012a). However, a precise analysis revealed that autophagy is absent in these cells in juvenile leeches. The midgut lumen in these specimens is filled with remnants of the embryonic nutritive material, so they do not feed themselves. Therefore, the juvenile specimens do not feed with blood, which could activate cell death. Starvation is a stress factor that activates autophagy (Park and Takeda 2008; Malagoli et al. 2010; Rost-Roszkowska et al. 2012b). However, juvenile specimens of *P. geometra* digest the embryonic nutritive material and the process of starvation does not appear and does not activate autophagy. In contrast, autophagy in *P. geometra* appeared in the adult specimens as a common process (80–90 % cells showed signs of autophagy) that was activated during and after feeding on blood in the digestive cells, and it was not dependent on the time after feeding (Table 2). *P. geometra* can survive for long periods without feeding; it usually feeds five to six times during its 6-month life (Bielecki, personal communication) like some other leeches, e.g., *Hirudo medicinalis* (Davies and McLoughlin 1996). Therefore, if we analyzed the specimens 1, 3, 7, and 14 days after blood feeding, starvation would not occur. Adult specimens after ~6 months of life copulate, and about 2 weeks after copulation, they lay cocoons and die. This can suggest that autophagy, which is strongly intensified just after feeding on blood, is not activated by starvation but is mainly involved in the protection of the epithelium cells against the cell death caused by damage to the organelles. The appearance of autophagy in several midgut digestive cells in non-feeding adults suggests that this process takes part in the neutralization of, e.g., damaged organelles like it does in many organisms. During this process, targeted organelles and/or proteins are separated from the entire cytoplasm within the autophagosomes (Levine and Klionsky 2004; Park et al. 2009, 2013; Malagoli et al. 2010). Therefore, we can conclude that if the toxic substances that originate from the blood affect the organelles, they are degraded due to autophagy. However, when cells do not cope successfully with toxic substances that originate from digestion, apoptosis in the form of cell death is activated (Giusti et al. 2007; Malagoli et al. 2010; Rost-Roszkowska et al. 2010b; Franzetti et al. 2012). The process of apoptosis observed in the midgut epithelium of the adult specimens intensifies with the time after feeding. Additionally, it was absent in non-feeding adults and adults that had been collected while feeding on the blood. Some apoptotic cells were observed after 1, 3, or 7 days after feeding, while in the specimens that were analyzed 14 days after feeding or after copulation, it occurred as a common process. This suggests that the products of blood digestion may cause the apoptosis of the digestive cells. This has also been suggested in blood-feeding insects when their organism is exposed to toxic haem activity (Okuda et al. 2007).

Another problem which occurred during our studies on cell death in *P. geometra* was the appearance of autophagy in all of the regions of the midgut (the esophagus, the crop, the PCC, and the intestine), while apoptosis was found only in the crop and PCC. We concluded that autophagy protects the digestive cells against damaged organelles and eventually delays the activation of cell death (Giusti et al. 2007; Rost-Roszkowska et al. 2010a). In addition, if the midgut epithelium is devoid of regenerative (embryonic) cells, the midgut epithelium must survive as long as possible. Degenerating organelles are separated from the cytoplasm and enclosed in autophagosomes thus preventing the activation of cell death (Rost-Roszkowska et al. 2012b). In our previous paper, we described sporadic small cells that appear between epithelial cells. However, their mitotic divisions and differentiation were not observed, so their regenerative role was not confirmed (Rost-Roszkowska et al. 2012a). Therefore, it is obvious that autophagy should be present in all regions of the digestive system which have contact with blood. However, in the case of apoptosis, we did not observe this process in the esophagus and intestine. We took into consideration the structure of the endodermal region of the digestive system of *P. geometra.* The crop and PCC are the longest and the most differentiated regions of the midgut with numerous large blind-end caeca and lateral diverticles (Rost-Roszkowska et al. 2012a), which enable the storage of blood for long periods. Therefore, their lengthy contact with blood and the products of blood digestion activates cell death. Similar results have been described in organisms which are exposed to toxic substances, e.g., metals that originate from the environment (Krug 2002; Amaral and Santos Rodrigues 2005; Wilczek et al. 2014). Thus, in the esophagus and intestine of the fish leech, the process of autophagy is sufficient to protect the epithelium, while in the long and differentiated crop and PCC, where cells do not as easily cope with toxic substance, apoptosis in the form of cell death is activated.

## Conclusions

Our studies show that (1) autophagy is a commonly observed process in the midgut epithelium of the species examined, which is intensified after the blood feeding; (2) autophagy is involved in the protection of the epithelium against damaged organelles and eventually the products of blood digestion; (3) apoptosis is activated by feeding on blood; and (4) the more sustained contact with the products of blood digestion, the more intensive the apoptosis that occurs.
